# Febrile infants risk score at triage (FIRST) for the early identification of serious bacterial infections

**DOI:** 10.1038/s41598-023-42854-z

**Published:** 2023-09-22

**Authors:** Shu-Ling Chong, Chenglin Niu, Gene Yong-Kwang Ong, Rupini Piragasam, Zi Xean Khoo, Zhi Xiong Koh, Dagang Guo, Jan Hau Lee, Marcus Eng Hock Ong, Nan Liu

**Affiliations:** 1 Department of Emergency Medicine, KK Women’s and Children’s Hospital, 100 Bukit Timah Road, Singapore, 229899 Singapore; 2 Paediatrics Academic Clinical Programme, Duke-NUS Medical School, 8 College Road, Singapore, 169857 Singapore; 3 Emergency Medicine Academic Clinical Programme, Duke-NUS Medical School, 8 College Road, Singapore, 169857 Singapore; 4 Present Address: Centre for Quantitative Medicine, Duke-NUS Medical School, 8 College Road, Singapore, 169857 Singapore; 5 KK Research Centre, KK Women’s and Children’s Hospital, 100 Bukit Timah Road, Singapore, 229899 Singapore; 6 Department of Paediatrics, KK Women’s and Children’s Hospital, 100 Bukit Timah Road, Singapore, 229899 Singapore; 7 Department of Emergency Medicine, Singapore General Hospital, 1 Hospital Crescent, Outram Road, Singapore, 169608 Singapore; 8 Children’s Intensive Care Unit, KK Women’s and Children’s Hospital, 100 Bukit Timah Road, Singapore, 229899 Singapore; 9 Health Services Research Centre, Singapore Health Services, 8 College Road, Singapore, 169857 Singapore

**Keywords:** Diagnostic markers, Infectious diseases, Paediatric research

## Abstract

We aimed to derive the Febrile Infants Risk Score at Triage (FIRST) to quantify risk for serious bacterial infections (SBIs), defined as bacteremia, meningitis and urinary tract infections. We performed a prospective observational study on febrile infants < 3 months old at a tertiary hospital in Singapore between 2018 and 2021. We utilized machine learning and logistic regression to derive 2 models: FIRST, based on patient demographics, vital signs and history, and FIRST + , adding laboratory results to the same variables. SBIs were diagnosed in 224/1002 (22.4%) infants. Among 994 children with complete data, age (adjusted odds ratio [aOR] 1.01 95%CI 1.01–1.02, *p* < 0.001), high temperature (aOR 2.22 95%CI 1.69–2.91, p < 0.001), male sex (aOR 2.62 95%CI 1.86–3.70, p < 0.001) and fever of ≥ 2 days (aOR 1.79 95%CI 1.18–2.74, *p* = 0.007) were independently associated with SBIs. For FIRST + , abnormal urine leukocyte esterase (aOR 16.46 95%CI 10.00–27.11, *p* < 0.001) and procalcitonin (aOR 1.05 95%CI 1.01–1.09, *p* = 0.009) were further identified. A FIRST + threshold of ≥ 15% predicted risk had a sensitivity of 81.8% (95%CI 70.5–91.0%) and specificity of 65.6% (95%CI 57.8–72.7%). In the testing dataset, FIRST + had an area under receiver operating characteristic curve of 0.87 (95%CI 0.81–0.94). These scores can potentially guide triage and prioritization of febrile infants.

## Introduction

Young infants with fever are at risk of bacteremia, meningitis and urinary tract infections (UTIs), collectively named serious bacterial infections (SBIs)^1^. The fear of missing SBIs has led to low physician thresholds to perform invasive investigations (including blood, urine and cerebrospinal fluid [CSF] cultures), resulting in a large number of unnecessary hospitalizations and rising healthcare costs^2^. Widespread empirical antibiotic use has also contributed to global antibiotic resistance^3^. Clinical prediction rules have thus far focused on identifying infants at low risk of SBIs who do not require extensive tests^1,4,5^. These clinical prediction rules have potential to reduce the number of invasive procedures^6,7^, but do not provide comprehensive guidance on which young febrile infant should be prioritized to receive urgent antibiotics. Such guidance is needed to reduce recognition delays and shorten time-to-antibiotics for infants who require urgent interventions^8^. Moreover, generalizability of these prediction rules has been questioned, with variable diagnostic performance in different populations^9,10^.

More recently, data-driven techniques including machine learning methods have been employed to derive and validate models to predict which young febrile infants are at risk of SBIs and invasive bacterial infections (IBIs)—namely meningitis and bacteremia^11,12^. These machine learning algorithms use commonly available triage information including age and temperature, and laboratory tests such as abnormal urinalysis, white blood cell, absolute neutrophil count (ANC), and procalcitonin to build scores that predict for the presence of SBI or IBI. Implementation of these algorithms could potentially reduce unnecessary lumbar punctures by approximately 70%^11^. However, these models are computationally complex and are not easily interpreted by clinicians^11^.

The AutoScore machine learning-based method, previously described as a combination of machine learning and logistic regression, automates the development of parsimonious and transparent risk models^13^. As compared to other machine learning methods, Autoscore has the potential to develop point-based scores that are interpretable by clinicians and can be translated into clinical practice. One example was the development and assessment of a Score for Emergency Risk Prediction (SERP) to estimate mortality after emergency admissions^14^.

We aimed to derive interpretable risk scores based on routinely available patient information and clinical data, to quantify risk of SBIs among infants < 3 months presenting with fever.

## Methods

### Study design and setting

We performed a prospective observational study for febrile infants < 3 months old presenting to a tertiary pediatric hospital in Singapore between December 2018 and December 2021. Our hospital is one of two pediatric hospitals in the country, with an annual ED attendance of about 150,000 children. Infants < 3 months old are routinely hospitalized in our institution. Neonates (defined as < 28 days old) receive the entire septic workup (blood, urine and CSF cultures) and proceed on to receive empirical antibiotics, while infants between 28 and 90 days’ old have variable investigations depending on the temperature trend and clinical assessment of the child. Regardless of the extent of investigations, infants < 3 months are monitored in the hospital until they are fever-free for 24 h, before discharge. We defined fever as an axilla or rectal temperature of 38 °C and above. Between December 2018 to December 2020, infants were recruited as part of a heart rate variability (HRV) study (NCT04103151). We subsequently obtained ethics approval to collect data from all febrile infants who presented between January 2021 and December 2021. We obtained approval from the SingHealth Institutional Review Board E in Singapore (2017/2680) with waiver of informed consent. The procedures used in this study adhere to the tenets of the Declaration of Helsinki.

### Data variables

We recorded patient demographics including age, birth gestation, and sex. We collected routine triage information including vital signs (i.e., temperature, heart rate, respiratory rate, and oxygen saturations). Our department uses the severity index score (SIS), a composite measure of respiratory effort, activity, color, play and temperature, to assess the acuity of a child at triage^15^. We obtained data on presence of comorbidities, duration of fever, and maternal Group B streptococcus (GBS) status. Laboratory investigations included hemoglobin, total white blood cell count, ANC, platelets, C-Reactive Protein (CRP), and procalcitonin. Fluid from urine and cerebrospinal fluid (CSF) were sent for analysis. Urine was tested for leukocyte esterase (graded as negative, 1 + , 2 + or 3 +) and nitrite (positive or negative). CSF was analyzed for cells and clarity. We obtained culture results from blood, urine and CSF. We recorded if the infant received fluid bolus resuscitation or inotropic support, intravenous antibiotics, or ventilator (both invasive and non-invasive) support. We also documented the need for high acuity care, defined as High Dependency (HD) and Intensive Care Unit (ICU) care.

### Outcome variables

SBI was defined as bacteremia, meningitis or UTI^1^. Bacteremia and meningitis were defined as pure growth of a pathogen in blood and CSF, respectively. When the bacteria grown was considered likely to be a contaminant (e.g., coagulase-negative staphylococcus), the case was not considered as SBI. UTIs were defined as growth of a single pathogen (a) > 100,000 colony-forming units (CFU/ml) in a clean catch specimen, or (b) ≥ 50,000 CFU/ml in a catheterized specimen, or (c) 10,000–50,000 CFU/ml in a catheterized specimen with an abnormal urinalysis (positive for leucocyte esterase or nitrite)^8^. We also recorded the duration of hospital stay. The study team members who recorded the outcome of SBI were not blinded to the clinical variables listed above.

### Statistical analysis

#### Data management

We described categorical variables using frequencies and percentages. Continuous variables were described using mean (and standard deviation, SD) or median (and interquartile range, IQR), depending on normality. Data were analyzed using R software, v 4.2.1 (R Foundation for Statistical Computing).

Because we wanted a practical tool to drive decision-making, we derived our score in 2 stages based on information that would be available to the ED physician. FIRST represents the initial triage phase and included patient demographics, vital signs and history-taking. FIRST + represents a more advanced phase after consultation and included laboratory investigations, such as urine and blood test results. Laboratory results would routinely require a turnaround time of up to 2 h, before becoming available to the ED physician. In our hospital, procalcitonin is part of the routine laboratory workup for hospitalized young infants with fever. For the group that physicians chose not to perform this blood test, we assigned them as ‘clinically not indicated’, rather than exclude them, because this reflected a group that yielded diagnostic information.

#### AutoScore machine learning-based method

To derive FIRST, we utilized the AutoScore technique. The AutoScore machine learning-based method has been described as a combination of machine learning and logistic regression, and automates the development of parsimonious risk models^13^. AutoScore consists of the following modules: variable transformation, variable ranking, score derivation and model selection, score fine-tuning, and model evaluation. The variable transformation module converts all continuous variables into categorical ones based on prespecified cutoffs. We transformed continuous variables to categorical variables with five categories based on 4 prespecified cutoffs, which included the 5% quantile, the 20% quantile, the 80% quantile, and the 95% quantile. The variable ranking module uses random forest to rank the variables based on their contribution to the outcome prediction. We used the ‘Parsimony Plot’ to show the predictive contribution of each variable. The score derivation module constructed a logistic regression model using the transformed variables, starting with the highest ranked variable and then adding on one variable at a time, following the order of their ranking. The study team selected the variables to be included in the final clinical score based on their domain knowledge and each variables’ contribution to the outcome prediction. We defined a significant correlation between two variables as having an absolute Pearson’s correlation coefficient of ≥ 0.2. Since such correlations would hinder accurate model fitting and prediction from subsequent logistic regression, only one variable from each significantly correlated variable group was selected into the subsequent analysis. The selected variables were used to build a multivariable logistic regression model, which was then converted to a clinical score. Study team members fine-tuned the resulting clinical score based on their domain knowledge for more practical and interpretable cutoff values. The final variables selected into the scoring models were fine-tuned according to clinical discretion and ease of use. Specifically, age was divided into only three groups: < 21 days, 21 to less than 28 days, and ≥ 28 days. Temperature was divided into four groups: < 38.5 °C, 38.5 °C to less than 39 °C, 39 °C to less than 40 °C, and ≥ 40 °C. Finally, the model evaluation module examined the out-of-sample prediction performance of the finalized clinical score. We presented the models’ performance on both the training and testing data, using area under receiver operating characteristic (ROC) curve, sensitivity, specificity, positive predictive value (PPV) and negative predictive value (NPV), as well as the model calibration results.

For sample size estimation, at a target sensitivity of 85%, marginal error of 0.05, and an event rate of 0.2 (based on published pilot data^8^), we aimed for a total of 980 febrile infants^16^. We did not perform multiple imputation for missing values. We only encountered missing values for laboratory values and detailed the number with complete data in the Results section.

#### Training and testing data

We divided our dataset into the training set and testing set. The training set was used for variable selection and score derivation, while the testing set was only used for model evaluation to examine the clinical score’s real-world performance on previously unseen data. The two sets were divided using the randomized stratified sampling, where the proportion of patients with SBI was the same in both sets. In this study, we used 80% and 20% of the available data for the training and testing set, respectively.

We followed the TRIPOD checklist for prediction model development^17^.

### Ethics approval

We obtained approval from the SingHealth Institutional Review Board E in Singapore (2017/2680) with waiver of informed consent. The procedures used in this study adhere to the tenets of the Declaration of Helsinki.

## Results

**Figure 1:**
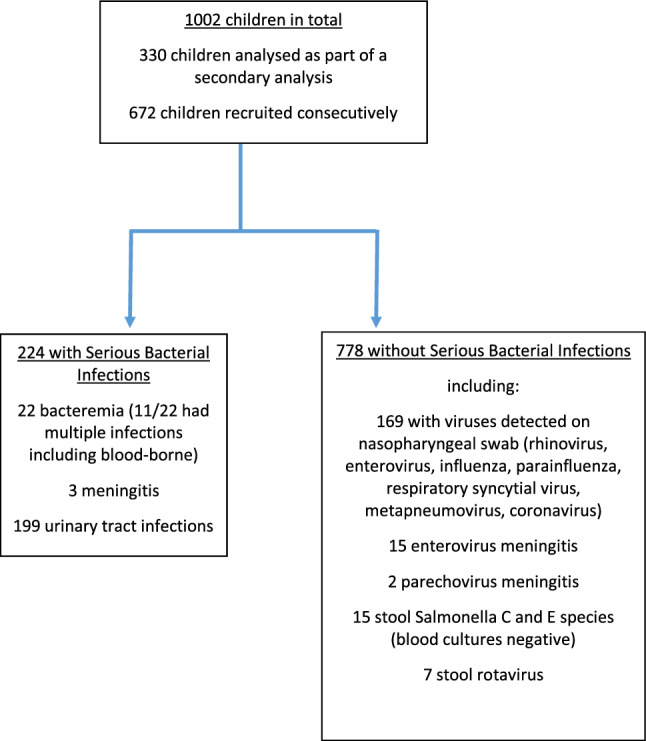
Flow diagram of patients included in the analysis.

We analyzed 1002 children in total, with an SBI rate of 22.4% (224/1002) (Fig. 1). The median age was 30 days (IQR 10–60) and there were 574 (57.3%) males. 741/1002 (73.9%) infants underwent urine cultures, 678/1002 (67.7%) blood cultures, and 477/1002 (47.6%) CSF cultures. Among infants with SBIs, the most common infection was UTIs (199/224, 88.8%), followed by bacteremia (22/224, 9.8%) and meningitis (3/224, 1.3%). Among the 22 infants with bacteremia, 11 (50.0%) had more than one source of SBI. The most common pathogens for UTIs (including co-infections) were *Escherichia ****c****oli* (153/199, 76.9%), *Klebsiella ****p****neumoniae* (30/199, 15.1%) and *Enterococcus ****f****aecalis* (12/199, 6.0%). Among the 22 infants with bacteremia, 7 (31.8%) had *GBS*, 7 (31.8%) had *Escherichia ****c****oli*, 2 (9.0%) had *Klebsiella ****p****neumoniae*. There were 2 patients (9.0%) who grew *Staphylococcus ****a****ureus* in both blood and urine cultures.

Infants with SBIs were older than those without SBIs (median age 46 days, IQR 21–69 vs. 28 days IQR 8–55, *p* < 0.001), and were more likely to be male (166/224, 74.1% vs. 408/778, 52.4%, *p* < 0.001) (Table 1). At triage, the presenting temperature and heart rate were significantly higher among infants with SBIs (38.7 °C SD 0.7 vs. 38.4 °C SD 0.5, *p* < 0.001 and 169 bpm SD 22 vs. 160 bpm SD 20, *p* < 0.001). Laboratory markers of inflammation (total white blood cell count, ANC, CRP, procalcitonin) were significantly higher among those with SBIs compared to those without (Table 1). There was no significant difference in the proportion of infants who received a fluid bolus between groups. However, infants with SBIs did experience a longer hospital stay compared to those without (median 4.0 days, IQR 3.0–5.0 vs. median 3.0 days IQR 2.0–4.0, *p* < 0.001). 6/224 (2.7%) of infants with SBIs and 14 /778 (1.8%) without SBIs required high acuity (HD and ICU) care (*p* = 0.407). One child required 10 days of non-invasive ventilation and inotropic support. This 31-day old infant had GBS bacteremia and meningitis, and had a prolonged hospital stay of 58 days. Data divided into training and testing sets are presented in Supplementary Tables 1 and 2.

**Table 1:** Patient characteristics and laboratory results and clinical management, stratified by presence of serious bacterial infections.

Variable	Serious bacterial infections (N = 224)	No Serious bacterial infections (N = 778)	*p* value
Age in days, median (IQR^a^)	46 (21–69)	28 (8–55)	** < 0.001**
Neonates (age < 28 days) (%)	60 (26.8%)	386 (49.6%)	** < 0.001**
Male sex (%)	166 (74.1)	408 (52.4)	** < 0.001**
Maternal GBS^b^ present/Known GBS^b^ status (%)	59/190 (31.1)	183/430 (29.9)	0.753
Temperature in °C	38.7 (0.7)	38.4 (0.5)	** < 0.001**
Heart rate, beats per minute	169 (22)	160 (20)	** < 0.001**
Respiratory rate, per min	42 (6)	42 (6)	0.311
Severity Index Score, median (IQR^a^)	9 (8–10)	9 (9–10)	** < 0.001**
Total white blood cells (× 10^9^/L)	N = 224	N = 703	** < 0.001**
	15.3 (6.0)	12.0 (4.8)	
Absolute neutrophil count (× 10^9^/L)	N = 224	N = 703	** < 0.001**
	7.9 (4.2)	4.9 (3.3)	
Hemoglobin (g/dL)	N = 224	N = 703	** < 0.001**
	12.3 (2.8)	14.0 (3.6)	
Platelet count (× 10^9^/L)	N = 224	N = 703	** < 0.001**
	458 (135)	417 (124)	
C-Reactive Protein, median (IQR^a^) (mg/L)	N = 199	N = 408	** < 0.001**
	33.5 (11.7–62.9)	5.9 (2.3–15.0)	
Procalcitonin, median (IQR^a^) (ng/mL)	N = 151	N = 429	**0.005**
	0.25 (0.10–2.25)	0.07 (0.05–0.15)	
Fluid bolus required	17 (7.6)	34 (4.4)	0.053
Length of hospital stay	4.0 (3.0–5.0)	3.0 (2.0–4.0)	** < 0.001**

^a^IQR = Interquartile range, ^b^GBS = Group B streptococcus. Continuous variables are expressed in mean (standard deviation, SD) unless stated otherwise.

Significant values are in [bold].

Based on 994 children with complete data, we built FIRST and FIRST + scores using the AutoScore pipeline. After variable transformation and correlation elimination (as per Methods), the selected candidate variables as well as the importance ranking for each variable are described in Supplementary Figs. 1 and 2. Candidate variables were then used for score derivation, which provided cross-validated parsimony plots shown in Supplementary Figs. 3 and 4. The correlation analyses for FIRST and FIRST + are found in Supplementary Figs. 5 and 6.

Based on the performance of each variable in the parsimony plots and clinical discretion of variable usability, we built the multivariable logistic regression model for SBIs in our cohort (Supplementary Table 3). In FIRST, age (aOR 1.01 95%CI 1.01–1.02, *p* < 0.001), high temperature (aOR 2.22 95%CI 1.69–2.91, *p* < 0.001), male sex (aOR 2.62 95%CI 1.86–3.70, *p* < 0.001) and fever of 2 or more days (aOR 1.79 95%CI 1.18–2.74, *p* = 0.007) were independently associated with SBIs. In FIRST + , all triage variables remained significant except for fever of 2 or more days. In addition, abnormal urine leukocyte esterase (aOR 16.46 95%CI 10.00–27.11, *p* < 0.001) and procalcitonin (aOR 1.05 95%CI 1.01–1.09, *p* = 0.009) were independently associated with SBIs (Supplementary Table 3). The calibration results and conversion plots for FIRST and FIRST + are detailed in Supplementary Figs. 7–10, while the decision curve analysis can be found in Supplementary Fig. 11.

The final risk scores selected for FIRST and FIRST + are presented in Table 2. Age, temperature, sex and day of fever were selected for FIRST. We found a U-shaped risk relationship for age. There was increased risk < 21 days old and ≥ 28 days, when compared to infants 21 to < 28 days old. Temperature had a linear relationship with likelihood of SBI, with increased risk scores assigned as the temperature increased. Children with 2 days or more of fever were more likely to have SBI. Urine leukocyte esterase and procalcitonin were further selected for FIRST + . The greater the abnormality in urine leukocyte esterase and procalcitonin, the higher the likelihood of SBI (Table 2).

**Table 2:** Risk score components in Febrile Infants Risk Score at Triage (FIRST and FIRST +).

Variable	Variable category	Points assigned
Febrile infants risk score at triage (FIRST) at triage		
Age	< 21 days	17
	21 to less than 28 days	0
	≥ 28 days	30
Temperature (°C)	< 38.5	0
	38.5 to less than 39.0	4
	39.0 to less than 40.0	13
	≥ 40.0	43
Sex	Female	0
	Male	17
Day of fever	< 2	0
	2 days or more	9
Febrile infants risk score at triage + (FIRST +) at triage and consultation		
Age	< 21 days	12
	21 to less than 28 days	0
	≥ 28 days	16
Temperature (°C)	< 38.5	0
	38.5 to less than 39.0	1
	39.0 to less than 40.0	6
	≥ 40.0	18
Sex	Female	0
	Male	9
Days of fever	< 2	-
	2 days or more	-
Urine leukocyte esterase	1 +	13
	2 +	17
	3 +	36
	Negative	9
	Clinically not indicated*	0
Procalcitonin (ng/mL)	< 0.05	0
	0.05 to less than 0.36	2
	0.36 to less than 4.38	8
	4.38 and above	21
	Clinically not indicated*	2

*This was determined by the attending Emergency Physician, based on a source of fever and clinical status.

**Figure 2:**
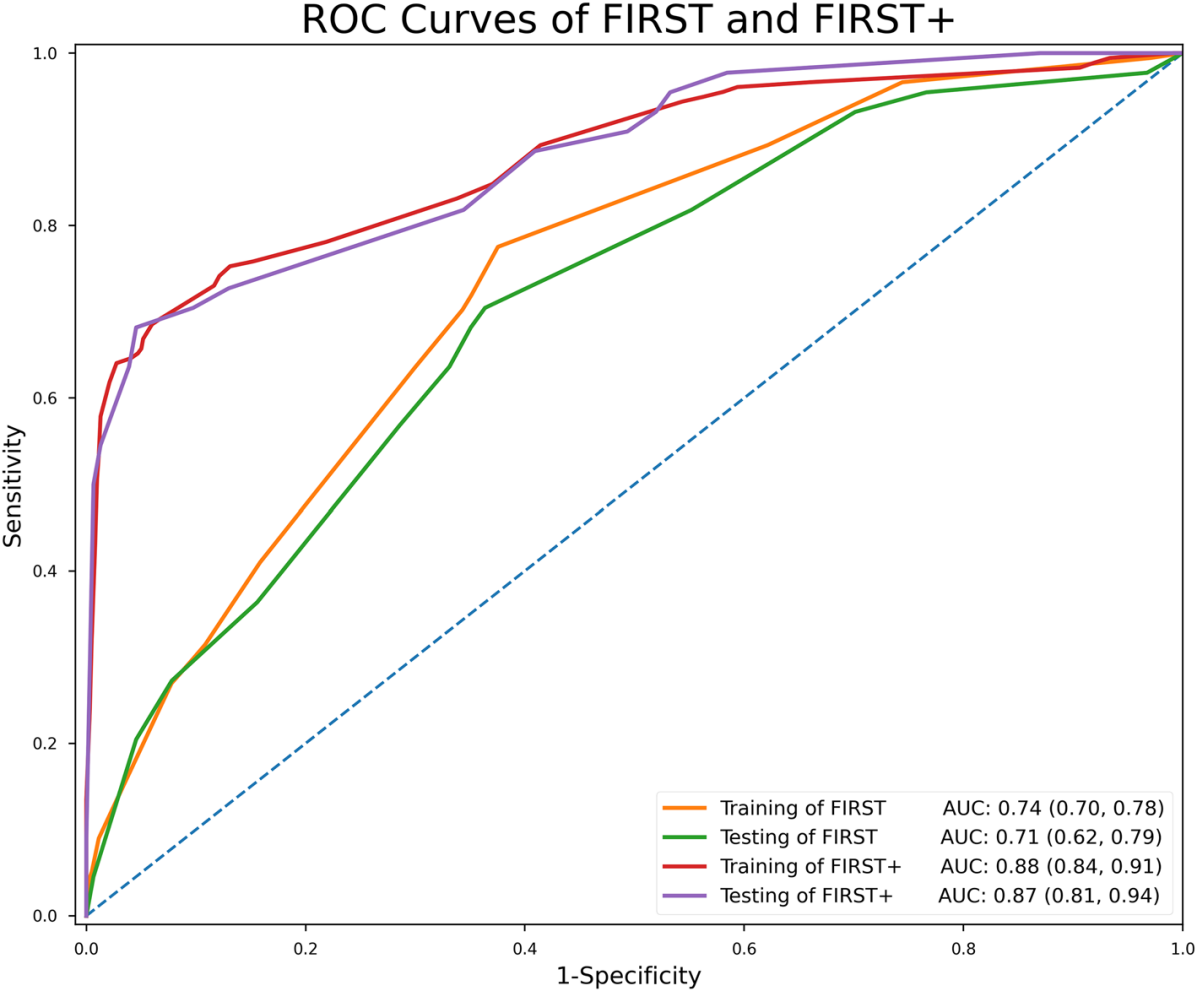
Area under receiver operating characteristic (ROC) curves of febrile infants risk score at triage (FIRST and FIRST +). *FIRST = Febrile Infants Risk Score at Triage. FIRST consists of Age, Temperature, Male Sex, Fever for 2 or more days FIRST + consists of Age, Temperature, Male Sex, Abnormal urine leukocyte esterase and procalcitonin.

Taking various thresholds, we present the proportion of patients who would test positive, and the corresponding performance of FIRST and FIRST + (Table 3). For example, at a FIRST threshold of ≥ 15% predicted risk (FIRST cut-off score ≥ 30), the model had a sensitivity of 93.2% (95%CI 84.1–100%), NPV of 94.0% (95%CI 86.3–100%), corresponding specificity of 29.9% (95%CI 22.7–37%) and would classify 75% of patients as high risk for SBI. When laboratory investigations are available, a FIRST + threshold of ≥ 15% predicted risk (FIRST + cut-off score ≥ 36) had a sensitivity of 81.8% (95%CI 70.5–91.0%), NPV of 92.7% (95%CI 88.2–96.5%), corresponding specificity of 65.6% (95%CI 57.8–72.7%) and classify 45% as high risk for SBI. The FIRST and FIRST + scoring models performed with a ROC of 0.71 (95%CI 0.62–0.79) and 0.87 (95%CI 0.81–0.94) on the testing set, respectively (Fig. 2).

**Table 3:** Sensitivity and specificity for various predicted risk thresholds.

Predicted Risk [> =]	Score cut-off [> =]	% of patients	Accuracy (95% CI)	Sensitivity (95% CI)	Specificity (95% CI)	PPV^a^ (95% CI)	NPV^b^ (95% CI)
Febrile infants risk score at triage (FIRST)							
1%	0	100	22.2% (22.2–22.2%)	100% (100–100%)	0% (0–0%)	22.2% (22.2–22.2%)	NA% (NA-NA%)
5%	13	97	23.7% (21.7–26.3%)	97.7% (93.2–100%)	2.6% (0.6–5.2%)	22.3% (21.2–23%)	80% (33.3–100%)
10%	21	81	39.4% (34.3–44.9%)	95.5% (88.6–100%)	23.4% (16.9–29.9%)	26.2% (24.2–28.4%)	95.1% (86.8–100%)
15%	30	75	43.9% (37.9–50%)	93.2% (84.1–100%)	29.9% (22.7–37%)	27.5% (24.8–30.4%)	94% (86.3–100%)
20%	38	44	65.2% (58.6–71.7%)	70.5% (56.8–84.1%)	63.6% (56.5–71.4%)	35.6% (29.3–42.4%)	88.4% (83.3–93.2%)
25%	43	40	66.2% (59.6–72.7%)	63.6% (47.7–77.3%)	66.9% (59.7–74.7%)	35.4% (28.4–43.4%)	86.6% (81.7–91.2%)
Febrile infants risk score at triage + (FIRST +) (triage and consultation)							
1%	16	96	26.3% (23.7–29.3%)	100% (100–100%)	5.2% (1.9–9.1%)	23.2% (22.6–23.9%)	100% (100–100%)
5%	27	75	46.5% (40.9–52%)	97.7% (93.2–100%)	31.8% (24.7–39%)	29.1% (26.9–31.4%)	98.1% (93.5–100%)
10%	33	52	65.7% (59.1–71.7%)	88.6% (79.5–97.7%)	59.1% (51.3–66.9%)	38.2% (33.6–43.8%)	94.9% (90.5–98.8%)
15%	36	45	69.2% (62.6–75.3%)	81.8% (70.5–91%)	65.6% (57.8–72.7%)	40.4% (34.4–47%)	92.7% (88.2–96.5%)
20%	39	24	84.8% (79.8–89.4%)	70.5% (56.8–84.1%)	89% (83.8–93.5%)	64.6% (53.7–76.7%)	91.3% (87.6–95.2%)
25%	41	24	85.4% (80.3–89.9%)	70.5% (56.8–84.1%)	89.6% (84.4–94.2%)	66% (54.5–77.5%)	91.4% (87.7–95.0%)
30%	43	20	88.4% (83.8–92.4%)	68.2% (54.5–81.8%)	94.2% (90.3–97.4%)	76.9% (65.1–89.5%)	91.2% (87.8–94.7%)

^a^PPV = Positive predictive value; ^b^NPV = Negative predictive value.

## Discussion

We studied 1002 febrile infants and reported an SBI rate of 22.4%, largely attributed to UTIs. Based on the Autoscore methodology, we derived FIRST, a triage predictive model that included age, temperature, sex and day of fever. We went on to derive and test FIRST + , based on availability of investigation results, and found that urine leukocyte esterase and procalcitonin were independently associated with SBIs. Adding on laboratory results improved the performance from a ROC of 0.71 (95%CI 0.62 – 0.79) (FIRST) to 0.87 (95%CI 0.81 – 0.94) (FIRST +) on the testing set.

The strength of our study is in the derivation and testing of an interpretable risk score. A previous supervised learning model for risk stratification of febrile infants acknowledged that it lacked parameter cutoffs and was computationally complex^11^. While machine learning models have promising performance compared to traditional scoring systems^12,18^, these have been difficult to translate to clinical practice because of the lack of recommended thresholds for action. In contrast, we assigned risk scores that quantified risk for SBI at each predictive risk threshold. Although our risk score requires refining and external validation, it can potentially guide clinical practice. Existing published clinical prediction rules have variable performance in different populations. A prior external validation of the PECARN rule in our population reported a sensitivity of 88.9%, specificity of 28.9%, and a ROC of 0.59 (0.42–0.76)^19^. These studies focus on identifying a group *at low risk* of SBI^1^, while our aim is to derive a tool that predicts for SBI, thereby serving as an adjunct to help clinicians prioritize which febrile infant requires urgent further investigations and management.

We reported the sensitivity and specificity at various thresholds (Table 3) to demonstrate how FIRST and FIRST + can aid clinicians to make informed decisions on disposition (hospitalization versus discharge), invasive investigations (including blood and CSF cultures), intervention (early antibiotics versus watchful waiting). These thresholds may vary based on physician practices and resource availability in different health services settings. For example, at the ED triage, most clinicians would favor a low threshold (one with a high sensitivity and NPV) to expedite care for the infant. A FIRST predictive threshold of 10% (score ≥ 21) with a sensitivity of 95.5% (95%CI 88.6–100%) and an NPV of 95.1% (86.8–100%) can prompt early consultation and close monitoring in the ED. Once these infants are examined and have initial laboratory investigations, clinicians may be willing to consider a higher threshold for action (FIRST +), one that has a higher specificity and will result in fewer infants subjected to more invasive tests. A major motivation would be to reduce unnecessary invasive blood and CSF tests. In this case, a FIRST + threshold of 15% (score ≥ 36) would have a higher specificity of 65.6% (95%CI 57.8–72.7%). In this case, 45% of infants would then be subject to further invasive investigations and empirical antibiotics.

We reported a higher prevalence of SBIs (22.4%) than another large multicenter cohort by the Pediatric Emergency Care Applied Research Network (PECARN) (9.3%)^1^. In the PECARN study, infants with clinical sepsis were excluded. However, clinician suspicion in this young infant population is notoriously inaccurate, hence we did not exclude these infants^20^. Also, our center is a pediatric tertiary institution (only one of two in the country) that receives walk-ins, as well as referrals from primary care and other hospitals. If the febrile infant was otherwise well with a known source of fever (e.g., respiratory symptoms likely secondary to an upper respiratory tract infection), the infant may have been managed in the primary care setting and not referred to our institution. Our findings must be interpreted in the context of this higher-than-expected SBI prevalence.

We found that the higher the temperature at triage, the more likely the febrile infant had an SBI, corresponding to higher risk scores. This is consistent with a retrospective cohort reported in our local population^21^. A multi-center study of 540 febrile infants similarly concluded that infants with IBIs had a higher median temperature compared to those without IBIs (38.8 °C vs. 38.4 °C)^22^. In our study, we only included infants who were febrile at triage. We did not account for infants who had fever at home but were afebrile at ED triage. It has been reported that infants who were afebrile at presentation to the ED but had fever at home had a significant risk of meningitis and other SBIs^23^. The presence and height of fever prior to ED attendance deserves further study.

The most common type of SBI in our study cohort was UTI, which is consistent with that of other study populations^1^. The diagnosis of UTI in a young febrile child can be challenging due to non-specific clinical presentation and the challenges associated with collecting a clean urine specimen^24^. In our study, we found that the presence of leukocyte esterase was independently associated with the presence of UTI. Besides leukocyte esterase, clinicians should also take into consideration the presence of nitrite in the urine dipstick, and urine white cell count when urinalysis results are available^25^. The large majority of febrile infants with UTIs were males (151/199, 81.2%), accounting for the male predominance in the SBI cohort (166/224,74.1%), overall. We did not have data on circumcision rates in this study cohort, and recognize that if circumcision rates were low, that may have contributed to the high UTI prevalence among male infants.

Procalcitonin has been widely reported to be useful in this population and has been included in risk stratification algorithms^1,4^. Procalcitonin has been reported as an early marker of infection, and elevated levels between 12 and 36 h of fever suggest the presence of an IBI in hospitalized febrile neonates ^26^. Our data-driven risk scores provided threshold cutoffs at 0.05, 0.36 and 4.38 (Table 2). Adding procalcitonin to a febrile infant clinical pathway resulted in decreased lumbar puncture for infants with at low risk of SBI^26^. However, more infants were assigned high risk and underwent laboratory investigations, resulting in no net change in overall resource utilization. This study highlights the need to study the implications of routine procalcitonin testing^7,27^.

We recognize the limitations to this study. Being a single-center study, the patient population is likely to differ from those of other centers, necessitating external validation and refinement of our risk score. We did not have culture results for all febrile infants (specifically infants 28–90 days old) because the decision on the extent of investigations was determined by the attending pediatrician. However, all febrile infants were hospitalized and monitored until 24 h afebrile before discharge, making a missed SBI less likely. Axillary temperature is the standard practice in our center, which is potentially confounded by over-wrapping and less accurate than rectal temperature. However, axillary temperature measurement is less invasive than rectal measurement, and the triage nurses are taught to take the axillary temperature with a single layer of clothing only. Our study dates included that of the COVID-19 pandemic when the presence of SARS-CoV-2 as well as the institution of national lockdowns could potentially have affected the prevalence of SBIs. The sensitivity of our model appears lower than some previously reported machine learning models^11,12^. However, in the FIRST methodology, all continuous variables are converted to categorical variables based on pre-specified cutoffs. This process of conversion likely reduced the prediction power of the continuous variables, accounting for a less ideal performance as compared to random forest models that applied continuous variables directly. Subsequent validation of this risk score must keep close surveillance on missed cases of SBI, quality of care and impact on health services^28^. Febrile infant populations vary depending on SBI prevalence, accessibility to care and healthcare-seeking behaviors. Some febrile infants may present within hours of the onset of fever, while others may present after the first day of fever. Therefore, risk scores need to be fine-tuned based on the unique characteristics of the local population.

## Conclusion

We derived and internally validated clinical risk scores (FIRST and FIRST +) for febrile infants that quantify the risk of SBIs using routinely available clinical predictors. If externally validated, this risk score has potential to guide triage and prioritization of febrile infants.

### Supplementary Information

Supplementary Figures.

Supplementary Table 1.

Supplementary Table 2.

Supplementary Table 3.

## Data Availability

The datasets generated during and/or analysed during the current study are not publicly available but are available from the corresponding author on reasonable request.

## Abbreviations

ANC: Absolute neutrophil count
CRP: C-reactive protein
CSF: Cerebrospinal fluid
ED: Emergency department
GBS: Group B streptococcus
HD: High dependency
IBI: Invasive bacterial infection
ICU: Intensive care unit
IQR: Interquartile range
NPV: Negative predictive value
PPV: Positive predictive value
ROC: Receiver operating characteristics
SBIs: Serious bacterial infection
UTI: Urinary tract infection
