# Supplementary material for: Febrile infants risk score at triage (FIRST) for the early identification of serious bacterial infections
Source: Sci Rep. 2023 Sep 22;13:15845. doi: 10.1038/s41598-023-42854-z (PMC10516995; doi:10.1038/s41598-023-42854-z)
Supplement: Supplementary file 2 — Supplementary Table 1. [file 41598_2023_42854_MOESM2_ESM.docx]

**Supplementary Table 1 (Training set).** **Patient characteristics and laboratory results and clinical management, stratified by presence of serious bacterial infections**

| **Variable** | **Serious bacterial infections (N=178)** | **No Serious bacterial infections (N=618)** | **p value** |
| --- | --- | --- | --- |
| **Age in days, median (IQR^a^)** | 45 (22 – 67) | 27 (8-55) | **<0.001** |
| **Neonates (age < 28 days) (%)** | 47 (26.4%) | 316 (51.1%) | **<0.001** |
| **Male sex (%)** | 132 (74.2) | 326 (52.8) | **<0.001** |
| **Temperature in ^o^C** | 38.6 (0.7) | 38.3 (0.5) | **<0.001** |
| **Heart rate, beats per minute** | 166 (22) | 160 (20) | **<0.001** |
| **Respiratory rate, per min** | 40 (6) | 40 (7) | 0.397 |
| **Severity Index Score, median (IQR^a^)** | 9 (8 – 10) | 9 (9 – 10) | **0.002** |
| **Total white blood cells (x10^9^/L)** | N=178  15.5 (6.1) | N=562  11.7 (4.9) | **<0.001** |
| **Absolute neutrophil count (x10^9^/L)** | N=178  7.46 (4.4) | N=562  4.3 (3.3) | **<0.001** |
| **Hemoglobin (g/dL)** | N=178  11.5 (2.6) | N=562  12.9 (3.6) | **<0.001** |
| **Platelet count (x10^9^/L)** | N=178  450 (135) | N=703  403 (122) | **<0.001** |
| **C-Reactive Protein, median (IQR^a^) (mg/L)** | N=178  27.7 (8.2 – 54.5) | N=555  1.4 (1.0 – 6.8) | **<0.001** |
| **Procalcitonin, median (IQR^a^) (ng/mL)** | N=178  0.25 (0.10 – 1.80) | N=555  0.07 (0.05 – 0.15) | **<0.001** |
