# Supplementary material for: Febrile infants risk score at triage (FIRST) for the early identification of serious bacterial infections
Source: Sci Rep. 2023 Sep 22;13:15845. doi: 10.1038/s41598-023-42854-z (PMC10516995; doi:10.1038/s41598-023-42854-z)
Supplement: Supplementary file 3 — Supplementary Table 2. [file 41598_2023_42854_MOESM3_ESM.docx]

**Supplementary Table 2 (Testing set).** **Patient characteristics and laboratory results and clinical management, stratified by presence of serious bacterial infections**

| **Variable** | **Serious bacterial infections (N=44)** | **No Serious bacterial infections (N=154)** | **p value** |
| --- | --- | --- | --- |
| **Age in days, median (IQR^a^)** | 54 (19 – 71) | 33 (9-62) | **0.02** |
| **Neonates (age < 28 days) (%)** | 13 (29.5%) | 66(42.9%) | **<0.001** |
| **Male sex (%)** | 33 (75.0) | 79 (51.3) | **0.008** |
| **Temperature in ^o^C** | 38.7 (0.7) | 38.3 (0.5) | **<0.001** |
| **Heart rate, beats per minute** | 171 (19) | 160 (21) | **<0.001** |
| **Respiratory rate, per min** | 40 (5) | 40 (5) | 0.625 |
| **Severity Index Score, median (IQR^a^)** | 9 (8 – 9) | 9 (9 – 10) | **<0.001** |
| **Total white blood cells (x10^9^/L)** | N=44  13.9 (5.0) | N=137  11.5 (4.4) | **0.003** |
| **Absolute neutrophil count (x10^9^/L)** | N=44  7.8 (3.4) | N=137  4.2 (3.3) | **<0.001** |
| **Hemoglobin (g/dL)** | N=44  11.5 (3.2) | N=137  12.4 (3.7) | **0.06** |
| **Platelet count (x10^9^/L)** | N=44  419 (138) | N=137  428 (130) | **<0.001** |
| **C-Reactive Protein, median (IQR^a^) (mg/L)** | N=44  28.9 (8.9 – 62.1) | N=136  1.6 (0.95 – 8.07) | **<0.001** |
| **Procalcitonin, median (IQR^a^) (ng/mL)** | N=27  0.24 (0.11 – 2.20) | N=81  0.08 (0.05 – 0.17) | **<0.001** |
