# Supplementary material for: Febrile infants risk score at triage (FIRST) for the early identification of serious bacterial infections
Source: Sci Rep. 2023 Sep 22;13:15845. doi: 10.1038/s41598-023-42854-z (PMC10516995; doi:10.1038/s41598-023-42854-z)
Supplement: Supplementary file 4 — Supplementary Table 3. [file 41598_2023_42854_MOESM4_ESM.docx]

**Supplementary Table 3. Univariate and multivariable logistic regression for Serious Bacterial Infections using the Febrile Infants Risk Score at Triage (FIRST and FIRST+)**

| **Variable** | **Unadjusted Odds Ratio (95%CI)** | **Unadjusted p value** | **Adjusted OR (95%CI) (FIRST)** | **Adjusted p value** | **Adjusted OR (95%CI) (FIRST+)** | **Adjusted p value** |
| --- | --- | --- | --- | --- | --- | --- |
| **Febrile Infants Risk Score at Triage (FIRST)** | | | | |  |  |
| **Age** | **1.02**  **(1.01 – 1.02)** | **<0.001** | **1.01**  **(1.01 – 1.02)** | **<0.001** | **1.01**  **(1.00 – 1.02)** | **0.017** |
| **Temperature reading** | **2.60**  **(2.00 – 3.37)** | **<0.001** | **2.22**  **(1.69 – 2.91)** | **<0.001** | **1.90**  **(1.26 – 2.86)** | **0.002** |
| **Male Sex** | **2.60**  **(1.87 – 3.61)** | **<0.001** | **2.62**  **(1.86 – 3.70)** | **<0.001** | **2.93**  **(1.76 – 4.89)** | **<0.001** |
| **Fever for 2 or more days** | **2.22**  **(1.50 – 3.30)** | **<0.001** | **1.79**  **(1.18 – 2.74)** | **0.007** | 1.08  (0.54 – 2.15) | 0.826 |
| **Febrile Infants Risk Score at Triage+ (FIRST+) (Triage and Consultation)** | | | | |  |  |
| **Abnormal Urine Leukocyte Esterase** | **11.40**  **(8.05 – 16.14)** | **<0.001** | **-** | **-** | **16.46**  **(10.00 – 27.11)** | **<0.001** |
| **Procalcitonin** | **1.09**  **(1.03 – 1.15)** | **0.002** | **-** | **-** | **1.05**  **(1.01 – 1.09)** | **0.009** |

***Cells with (-) are not applicable for FIRST**
