# Supplementary material for: Febrile infants risk score at triage (FIRST) for the early identification of serious bacterial infections
Source: Sci Rep. 2023 Sep 22;13:15845. doi: 10.1038/s41598-023-42854-z (PMC10516995; doi:10.1038/s41598-023-42854-z)
Supplement: Supplementary file 1 — Supplementary Figures. [file 41598_2023_42854_MOESM1_ESM.docx]

**Supplementary Figure 1 Selected Variables for Febrile Infants Risk Score at Triage (FIRST) and Their Rankings**

SpO2= Pulse oximetry; SIS=Severity Index Score; GBS=Group B Streptococcus; PROM=Premature Rupture of Membranes

**Supplementary Figure 2 Selected Variables for Febrile Infants Risk Score at Triage + (FIRST+) and Their Rankings**

UFEME=Urine Full and Microscopic Examination; SpO2= Pulse oximetry; SIS=Severity Index Score; GBS=Group B Streptococcus; PROM=Premature Rupture of Membranes

**Supplementary Figure 3 Parsimony Plot of Area under Receiver Operating Characteristics (ROC) for Variables selected for Febrile Infants Risk Score at Triage (FIRST)**

SpO2= Pulse oximetry; SIS=Severity Index Score; GBS=Group B Streptococcus; PROM=Premature Rupture of Membranes

The number in each bar refers to the variable ranking (refer Supplementary Figure 1)

**Supplementary Figure 4 Parsimony Plot of Area under Receiver Operating Characteristics (ROC) for Variables selected for Febrile Infants Risk Score at Triage + (FIRST+)**

UFEME=Urine Full and Microscopic Examination; SpO2= Pulse oximetry; SIS=Severity Index Score; GBS=Group B Streptococcus; PROM=Premature Rupture of Membranes

The number in each bar refers to the variable ranking (refer Supplementary Figure 2)

**Correlation Analysis**

For the correlation analysis of variables used to develop FIRST, the correlations between the initial variables can be found in the plot below.

**Supplementary Figure 5. Correlation Analysis for Febrile Infants Risk Score at Triage (FIRST)**

Based on the correlation coefficients and our clinical knowledge, we included the following variables: ‘Age in days’, ‘Sex’, ‘Temperature’, ‘Respiratory_Rate’, ‘SpO2’, ‘SIS’ (Severity index score), ‘Prematurity’, ‘Comorbidities’, ‘PROM’ (Premature rupture of membranes), ‘GBS’ (Group B Streptococcus), ‘Prior Perinatal Sepsis’, and ‘Day of Fever’

For the development of FIRST+, the correlations between the initial candidate variables can be found in the plot below. The variables excluded for FIRST were also excluded at the beginning of FIRST+ development.

**Supplementary Figure 6. Correlation Analysis for Febrile Infants Risk Score at Triage + (FIRST+)**

As the correlation plot shows, a lot of the categorized blood tests results are highly correlated with each other. Therefore, the variables we selected to go through the subsequent analysis are as follows: ‘Age in days’, ‘Sex’, ‘Temperature’, ‘Respiratory_Rate’, ‘SpO2’, ‘SIS’ (Severity index score), ‘Prematurity’, ‘Comorbidities’, ‘PROM’ (Premature rupture of membranes), ‘GBS’ (Group B Streptococcus), ‘Prior Perinatal Sepsis’, and ‘Day of Fever’, ‘Coded Procalcitonin’, and ‘UFEME leukocyte esterase’.

**Calibration results of FIRST and FIRST+**

It is worth noting that the scores given by AutoScore does not represent the actual probabilities estimated by the underlying logistic model. To ensure the scores provided by AutoScore are all positive (i.e., being a positive integer value such as 1 or 5), the original model fitted on the data were modified based on that with the lowest model coefficient as the new reference. Therefore, the relationship between a score and the underlying predicted risk is logit. More details on this transformation can be found in the original AutoScore paper.

The calibration plots we provide here are all based on the initial underlying models used in AutoScore without the change of reference. We also provide conversion plots (in the decision curve analysis section) to show the relationship between the FIRST or FIRST+ scores and the predicted risks.

The calibration plot for FIRST is shown below. The calibration slope is 0.834, while the intercept being -0.104. We notice that the ROC-AUC of the calibration is slightly higher than the one given in the testing of FIRST. The reason for this is the fact that AutoScore rounds up the coefficient in the logistic model to produce integer scores for better usability and interpretability.

**Supplementary Figure 7. Correlation Analysis for Febrile Infants Risk Score at Triage (FIRST)**

The calibration plot of FIRST+ is as below. The calibration slope is 0.993 with the intercept being 0.145. We also notice the slight increase of AUC in the calibration, as we explained earlier.

**Supplementary Figure 8. Correlation Analysis for Febrile Infants Risk Score at Triage+ (FIRST+)**

To illustrate the relationship between Scores from AutoScore and the predicted risk from the underlying models, we provide the risk conversion plots from scores to predicted risks for both FIRST and FIRST+. Table 3 in the manuscript also provides conversions to scores based on some of the commonly used risk cut-off values. The conversion plot for FIRST is shown below.

**Supplementary Figure 9. Conversion Plot for Febrile Infants Risk Score at Triage (FIRST)**

The conversion plot for FIRST+ is shown as below:

**Supplementary Figure 10. Conversion Plot for Febrile Infants Risk Score at Triage+ (FIRST+)**

**Decision curve analysis**

The decision curve analysis (DCA) results shown in this section were based on the underlying logistic models without of change of reference. The DCA plots of FIRST and FIRST+ can be found in the figure below.

**Supplementary Figure 11. Decision Curve Analysis**
